# Validation of a German version of the caregiver strain questionnaire-short form 11 (CGSQ-SF11)

**DOI:** 10.1186/s40359-024-01875-7

**Published:** 2024-07-10

**Authors:** Julia M. Göldel, Petra Warschburger

**Affiliations:** Department of Psychology, Counseling Psychology, University of Potsdam, Karl-Liebknecht-Str. 24-25, 14476 Potsdam, Germany

**Keywords:** Caregiver strain, Special healthcare needs, Parents, Validation, Psychometrics

## Abstract

**Objective:**

Caring for a child, particularly one with special healthcare needs, is a demanding task that can lead to the experience of caregiver strain. This in turn has an effect on the caregiver’s mental health, as well as on the child and his or her treatment. To enable the identification of afflicted parents, this study aims to provide a German version of the Caregiver Strain Questionnaire–Short Form 11 (CGSQ-SF11) and to examine its factor structure and psychometric properties.

**Methods:**

Data from 698 caregivers were included in the analyses. Caregivers completed the CGSQ-SF11 along with measures of parenting stress (PSI-SF), stress (PSS-10), anxiety (GAD-7), depression (PHQ-8), family-related quality of life (FLQ), and social desirability (SES-17) as additional instruments for validation. A two-week follow-up questionnaire included only the CGSQ-SF11. Exploratory factor analysis followed by a confirmatory factor analysis was conducted for parents of children with and without special healthcare needs, separately. Further analyses examined the validity and reliability of the instrument.

**Results:**

For parents of children with special healthcare needs, a three-factor structure (objective, internalized subjective, externalized subjective strain) with a second-order factor (caregiver strain) was supported. For parents of children without special healthcare needs, a similar three-factor structure was found, although the second-order factor was not supported. Measurement invariance between the two groups was not confirmed. Internal consistency, test-retest reliability, and validity were largely supported in both groups.

**Conclusions:**

The results indicate that the German version of the CGSQ SF-11 is a valid and reliable questionnaire for measuring caregiver strain.

**Supplementary Information:**

The online version contains supplementary material available at 10.1186/s40359-024-01875-7.

## Background

Children’s development, experiences, and emotions are to a large extent influenced by their caregivers. The caregiving role is therefore one of great responsibility and is accompanied by multiple stressors, particularly for parents of children with special healthcare needs (SHCN), including chronic health conditions and mental health problems [1]. The parents of these children play a crucial role in their child’s long-term care, for example by organizing medical appointments, implementing medical measures into everyday life, and providing emotional support [2]. This can lead to caregiver strain (CS). However, caring for a typically developing child can also be demanding and give rise to CS [3, 4].

In line with Brennan, Babinski, and Waschbusch [5], CS refers to the demands and negative emotional consequences associated with childcare that exceed normal caregiving demands. Objective CS refers to the observable negative consequences, including direct and indirect care tasks, the demands of the child’s emotional needs, and the impact on family life. Subjective CS, on the other hand, comprises the caregiver’s resulting negative emotions [6], that can be inwardly directed (internalized subjective strain) or outwardly directed (externalized subjective strain) [1, 5].

The experience of CS has multiple consequences for both the parent and the child. With respect to the parents, increased CS is associated with deteriorated mental health [3, 7] and less satisfaction with life [8]. Afflicted parents are more likely to engage in less effective parenting practices and to report impaired parent-child relationships [1, 4]. With respect to the child, CS is accompanied by poorer mental health [9] and increased symptoms of illness [10]. Furthermore, disease management is influenced as well: parents experiencing CS have more difficulties in communicating and organizing treatment appointments. As a result, they are more likely to utilize mental health services, and generate higher healthcare costs [11].

To prevent a vicious cycle of CS and the child’s symptoms and to provide suitable support, it is vital to identify parents with increased CS. In German-speaking countries, there is currently no instrument available to measure CS in an economic and multidimensional way. Parenting stress questionnaires refer to general parental burdens without taking into account burdens that go beyond regular caregiving. One exception is the Impact on Family Scale (FaBel; [12]). However, due to its wording, it can only be applied to parents of children with chronic physical health conditions and is not designed for mental health problems, further SHCN or for healthy children. Additionally, the Zarit Burden Interview (ZBI; [13]) can be employed, but it does not allow the differentiation between objective and subjective strain.

With the Caregiver Strain Questionnaire-Short Form 11 (CGSQ-SF11; [5]), a promising English-language questionnaire, is available. It is the shortened version of the Caregiver Strain Questionnaire (CGSQ; [1]), a commonly used measure to assess the burden on parents caring for a child with emotional or behavioral disorders, but also for parents of children without SHCN. The CGSQ consists of three subscales: objective, internalized subjective, and externalized subjective strain. In line with economic aspects, there are some short forms derived from the original 21-item scale [1, 5, 14]. Although the CGSQ-SF11 is not the shortest version, it has two distinct advantages: (1) While the other short forms omit the subjective externalized strain dimension, the CGSQ-SF11 consists of all three subscales. (2) To assess present CS rather than that CS experienced during salient previous periods, the CGSQ-SF11 uses the present tense and therefore enables researchers to capture trajectories over time. In addition, the item wording allows universal use across all caregiving parents. The three-factor structure of the CGSQ has been confirmed in several studies [1, 15–17], and was also found for the CGSQ-SF11 [5]. Also, a global score is often assumed [18]. In terms of convergent validity, the CGSQ-SF11 has demonstrated positive relationships with child psychopathology and impairment variables [5]. Furthermore, CGSQ scores are positively associated with mental health impairment and parenting stress, and negatively associated with caregiver life satisfaction, health-related quality of life, maladaptive coping, family functioning, and social support [1, 15, 19, 20]. Internal consistency of the CGSQ-SF11 ranges between Cronbach’s α = 0.88 − 0.96. Measurement invariance is confirmed for child and caregiver sex and age, and child psychopathology variables [5].

We aimed to provide a psychometrically sound German version of the CGSQ-SF11 for use in parents of children with SHCN as well as parents of children without SHCN. To explore the factorial structure of the translated questionnaire, we conducted an exploratory factor analysis (EFA) in the first step, and a confirmatory factor analysis (CFA) in the second step of this study. To examine convergent and divergent validity, we focused on related and distinct constructs. A body of literature shows relations between CS and parenting stress [e.g., 20], stress [e.g., 21], anxiety and depression [e.g., 1, 7], and family-related quality of life [e.g., 22]. Furthermore, the measure of CS should not be affected by social desirability. Therefore, we assumed moderate to strong associations with parenting stress, stress, family-related quality of life, anxiety, and depression, as well as low correlations with social desirability. We hypothesized internal consistency and test-retest reliability.

## Methods

### Translation process

Following the WHO guidelines [23], the original CGSQ-SF11 was first translated into German by two independent psychologists. Discrepancies were discussed by a panel of experts in the field, also consisting of psychologists. This pre-final version was then back-translated by a bilingual person. To ensure comprehensibility, a pilot group of parents (*N* = 5) completed the questionnaire and confirmed understandability. The Questionnaire is provided in Additional File 1.

### Procedure

Data were collected between December 2021 and August 2022 using the online software Umfragen.up [24]. Recruitment took place via posts on social media platforms, online forums for parents, and flyer dissemination. Inclusion criteria were informed consent, a minimum age of 18 years, having a child up to 21 years of age (the age limit for transition from pediatric to adult care), and completion of at least one item of the CGSQ-SF11.

Participants filled in the online questionnaire after providing their informed consent and were invited to take part in a follow-up survey two weeks later. As an incentive to participate, a booklet with information about parenting in challenging times was provided. The study was approved by the institutional review board (reference number 65/2021).

### Sample characteristics

In total, 1091 caregivers participated in the study. Of these, 32 participants refused consent, 101 participants had children older than 21 years, and 259 participants did not fill in at least one item of the CGSQ-SF11. Due to indications of double participation, the data of one participant were removed.

Data from 698 participants were included in the final analysis. Using the CSHCN-Screener [25], we created two subsamples: 421 (60.32%) caregivers of children with SHCN were assigned to the patient group, while 277 (39.68%) caregivers whose children did not meet the criteria for SHCN were assigned to the healthy group. Table 1 summarizes the sample characteristics in both groups. Significant differences were found for parental sex (χ^2^(2) = 8.66, *p* = .01), age (t(598) = -8.09, *p* < .001), socioeconomic status (t(641) = 2.94, *p* = .003), and child age (t(92) = -11.32, *p* > .001).

**Table 1:** Sample description

Characteristic	Patient group	Healthy group	Test-Retest Subsample: Patient group	Test-Retest Subsample: Healthy group
N	421	277	204	122
Caregiver’s sex, n (%)				
Female	400 (95.01)	248 (89.86)	197 (96.57)	110 (90.16)
Male	18 (4.28)	27 (9.78)	5 (2.45)	12 (9.84)
Non-binary	3 (0.71)	1 (0.36)	2 (0.98)	0 (0.0)
Caregiver’s age^1^, M (SD)	41.94 (7.01)	37.60 (6.88)	42.46 (6.95)	37.8 (7.05)
Relation to child, n (%)				
Biological parent	393 (93.35)	264 (95.31)	185 (90.69)	115 (94.26)
Adoptive, foster, or step parent	26 (5.23)	11 (3.97)	19 (9.31)	7 (5.74)
Grandparent	1 (0.24)	0 (0.0)	0 (0.0)	0 (0.0)
Other	1 (0.24)	2 (0.72)	0 (0.0)	0 (0.0)
Family status				
Single-parenting, n (%)	64 (15.20)	31 (11.19)	24 (11.76)	9 (7.38)
In a relationship, n (%)	376 (89.31)	257 (92.78)	189 (92.65)	115 (94.26)
Socio-economic status, M (SD)	6.05 (1.67)	6.41 (1.46)	6.1 (1.54)	6.57 (1.39)
Child’s sex, n (%)				
Female	161 (38.24)	128 (46.21)	83 (40.69)	48 (39.34)
Male	258 (61.28)	149 (53.79)	121 (59.31)	74 (60.66)
Non-binary	2 (0.48)	0 (0.0)	0 (0.0)	0 (0.0)
Child’s age^1^, M (SD)	9.68 (4.71)	5.57 (4.69)	9.88 (4.61)	5.4 (4.62)

^1^ in years

The patient group comprises 99 (23.52%) caregivers of children with chronic physical health conditions, 160 (38.0%) caregivers of children with emotional or behavioral disorders, and 142 (33.73%) caregivers of children with both.

The sample of participants completing the follow-up questionnaire included 204 and 122 caregivers in each group.

### Measures

In addition to self-reported sex, age, and relationship status, the MacArthur Scale [26] was used to assess the subjective socioeconomic status.

#### Caregiver strain

The translated German version of the CGSQ SF-11 was applied to assess CS (e.g., “Do your child’s problems interrupt your personal time?”). Responses were recorded on a 5-point Likert Scale ranging from 1 “not at all” to 5 “very much”.

#### Parenting stress

Parenting Stress was assessed using an unpublished German version of the Parenting Stress Index (PSI-SF; [cf. 27]). In 33 of the 36 items (e.g., “My child is much more active than I expected”), answers are recorded on a 5-point Likert Scale ranging from 1 “strongly disagree” to 5 “strongly agree”; in three items, an answer can be chosen from 5 individual answer categories. In the present study, Cronbach’s α was 0.93 in the patient group and 0.94 in the healthy group.

#### Stress

To assess the caregiver’s stress, the German version of the Perceived Stress Scale-10 (PSS-10; [28]) was applied. Responses to its 10 items (e.g., “In the last month, how often have you felt nervous and stressed?”) are recorded on a 5-point Likert-scale ranging from 1 “never” to 5 “very often”. In previous research, the internal consistencies ranged between Cronbach’s α = 0.79 and 0.89 [28]. In the present study, Cronbach’s α was 0.87 in the patient group and 0.88 in the healthy group.

#### Family-related life quality

The family-related life quality questionnaire (FLQ; [22]) was applied to measure the caregiver’s quality of life. The 18 items (e.g., “Have you received any support from your partner?”) include statements on family life in the last week and are answered on a 5-point Likert Scale ranging from 1 “never/almost never” to 5 “very often”. The FLQ yielded internal consistencies between Cronbach’s α = 0.88 and 0.94 [22]. In the present study, Cronbach’s α was 0.92 in the patient group and 0.93 in the healthy group.

#### Anxiety

Anxiety was assessed using the German version of the Generalized Anxiety Disorder Screener (GAD-7; [29]) with 7 items assessing the frequency of anxiety symptoms in the last two weeks (e.g., “not being able to stop or control worrying”). The 4-point Likert Scale ranges from 0 “not at all” to 3 “nearly every day”. In previous research, Cronbach’s α was 0.89 [29]. In the present study, Cronbach’s α was 0.90 in the patient group and 0.88 in the healthy group.

#### Depression

The German version of the Patient Health Questionnaire PHQ-8 (i.e., PHQ-9 without item on suicidality; [30, 31]) was applied to measure depressive symptoms during the past two weeks (e.g., “feeling down, depressed, or hopeless”). Responses are recorded on a 4-point Likert scale ranging from 0 “not at all” to 3 “nearly every day”. The PHQ-9 has displayed a satisfactory internal consistency of Cronbach’s α = 0.88 [32]. In the present study, Cronbach’s α of the PHQ-8 was 0.81 in the patient group and 0.84 in the healthy group.

#### Social desirability

To assess the participants’ social desirability, the German version of the Social Desirability Scale-17 (SES-17; [33]) was applied. The 17 items include statements of positively and negatively connoted behavior patterns (e.g., “I never hesitate to stand by someone in need”), and are answered with a dichotomous response format (“yes” / “no”). In previous research, Cronbach’s α ranged between 0.72 and 0.75 [33]. In the present study, Cronbach’s α was 0.69 in the patient group and 0.66 in the healthy group.

### Statistical analyses

All analyses were performed using R Statistical Software (Version 4.2.3; [34]). In line with a universalist perspective [35, 36], we followed a two-step analytic approach: First, we conducted an EFA to explore the factor structure of the questionnaire. Subsequently, a CFA was performed to confirm the detected structure. To avoid circularity, we conducted both analyses separately by splitting the patient and healthy groups into two random subsamples each for EFA (n_patient group_ = 210; n_healthy group_ = 138) and CFA (n_patient group_ = 211; n_healthy group_ = 139). The subsamples did not differ significantly in key demographics except for the caregiver’s sex in the healthy group (*p* = .03; EFA: 118 female, 19 male, 1 non-binary; CFA: 130 female, 8 male). Missing data were estimated using the full information maximum likelihood (FIML) method [37].

The factorability of our data was tested using Bartlett’s test of sphericity, the Kaiser-Meyer-Olkin measure of sampling adequacy (KMO), and the determinant of the correlation matrix [36, 38].

As normal distribution was not given (see Table 2), the EFA was conducted using principal axis factoring (PAF) with oblique rotation, as correlated subscales were assumed. The number of factors was determined via Scree plot, Horn’s parallel analysis, Velicer’s minimum average partial test, and the Bayesian Information Criterion (BIC) [39, 40].

**Table 2:** Descriptive statistics for patient group and healthy group: EFA

Item		Patient group								Healthy group						
		M (SD)		Range		Skewness		Kurtosis		M (SD)		Range		Skewness		Kurtosis
1. interruption of time		3.79 (1.06)		4		-0.60		-0.57		2.75 (1.31)		4		0.08		-1.25
2. missing work		3.08 (1.19)		4		-0.06		-0.80		2.08 (1.18)		4		0.74		-0.62
3. financial strain		2.16 (1.28)		4		0.77		-0.64		1.17 (0.49)		3		3.27		11.28
4. disruption of relationships		2.48 (1.19)		4		0.43		-0.64		1.74 (1.02)		4		1.38		1.37
5. sad		3.51 (1.15)		4		-0.50		-0.57		2.11 (1.19)		4		0.94		-0.03
6. embarrassed		1.69 (1.03)		4		1.44		1.26		1.37 (0.72)		3		1.81		2.28
7. angry		1.84 (1.03)		4		1.09		0.37		1.90 (0.91)		3		0.56		-0.84
8. worried		3.86 (1.15)		4		-0.61		-0.76		2.19 (1.15)		4		0.62		-0.57
9. resentful		1.34 (0.67)		3		1.90		2.66		1.21 (0.52)		3		2.72		7.78
10. tired		3.68 (1.14)		4		-0.67		-0.35		2.64 (1.33)		4		0.35		-1.09
11. toll on family		3.33 (1.11)		4		-0.39		-0.71		2.15 (1.07)		4		0.59		-0.65

Based on the results of the EFA, a CFA was performed using the *lavaan* package. In addition, we tested the expected three-factor model. We also considered a second-order model with a global factor and all subscales. Due to the lack of a normal distribution, maximum likelihood estimation with robust (Huber-White) standard errors and a scaled test statistic (asymptotically) equal to the Yuan-Bentler test statistic (MLR) was applied [41]. In line with the requirements of factor analyses, bounds for variances and factor loadings were computed in the hierarchical models. Model fit was evaluated using the following goodness-of-fit indices: the robust chi-square χ^2^ (good: 0 ≤ *χ*^2^ ≤ 2*df*, acceptable: 2*df* < *χ*^2^ ≤ 3*df*), *p* (good: 0.05 < *p* ≤ 1.0, acceptable: 0.01 ≤ *p* ≤ 0.05), the robust comparative fit index (CFI; good: 0.97 ≤ CFI ≤ 1.0, acceptable: 0.95 ≤ CFI ≤ 0.97), the robust root mean square error of approximation (RMSEA; good: 0 ≤ RMSEA ≤ 0.05, acceptable: 0.05 < RMSEA ≤ 0.08), and the robust standardized root mean square residual (SRMR; good: 0 ≤ SRMR ≤ 0.05, acceptable: 0.05 < SRMR ≤ 0.10) [42]. To compare the models with a good or acceptable fit, *χ*^2^ difference tests were conducted [42]. RMSEA is presented with its 90% confidence interval.

A multigroup-CFA was considered to examine the applicability of the CGSQ-SF11 to parents of both children with and without SHCN. Measurement invariance was interpreted by *χ*^2^, as well as ΔCFI, ΔRMSEA, and ΔSRMR. Following Chen et al. [43], ΔCFI ≤ 0.005, ΔRMSEA ≤ 0.010, and ΔSRMR ≤ 0.025 (configural invariance) or ΔSRMR ≤ 0.005 (metric and scalar invariance) were used to indicate measurement invariance.

Cronbach’s α (excellent: α ≥ 0.90, good: 0.80 ≤ α ≤ 0.89, adequate: 0.70 ≤ α ≤ 0.79; [44]) and McDonald’s ω (should not be significantly lower than 0.70; [45]) were calculated to assess internal consistency. Test-retest reliability over two weeks was determined by intraclass correlation coefficients (ICC) using a two-way mixed effects model (poor: ICC < 0.5, moderate: 0.5 < ICC < 0.75, good: 0.75 < ICC < 0.9, excellent: > 0.9; [46]). To examine convergent and divergent validity, we conducted Pearson correlations (small effect size: *r* > 0.10, medium: *r* > 0.30, large: *r* > 0.50; [47]). Using the *measureQ* package, we also calculated the average variance extracted (AVE), which should be not significantly less than 0.50 and not significantly less than the squared correlations between two factors [45], the maximum shared variance (MSV), and the average shared variance (ASV), which should be smaller than the AVE [48]. In terms of incremental validity, hierarchical regression analyses were computed to determine whether CS explains additional variance in stress, anxiety, depression, and family-related quality of life beyond demographic variables and parenting stress. Caregiver’s and child’s age and sex, socioeconomic status, and parenting stress were included in the first step, and CGSQ-SF11 scores in the second step as predictors for each criterion variable.

Floor and ceiling effects were examined by focusing on the percentage of caregivers with the highest or lowest possible scores on a subscale. In accordance with McHorney and Tarlov [49], a limited ability to discriminate between participants was assumed when a cut-off of 15% was exceeded.

To check for common method bias, we conducted Harman’s single factor test (including the PSI-SF, PSS-10, FLQ, GAD-7, PHQ-8, SES-17, and CGSQ-SF11; values > 50% indicating common method bias) and controlled for an unmeasured latent method factor by comparing a model with all measures as indicators and a latent method factor with an equivalent model without the latent method factor [50].

## Results

### Acceptance of the CGSQ-SF11

There were no missing data within the CGSQ-SF11 in the patient group, whereas 0.36–1.81% of the data for each variable were missing in the healthy group.

### EFA – patient group

Means, standard deviations, range, skewness, and kurtosis are presented in Table 2. Although the items showed some skewness and kurtosis, the values were within the critical thresholds [51].

Bartlett’s test of sphericity (*χ*^2^(55) = 972.07, *p* < .001), the KMO measure (MSA = 0.85), and the determinant of the correlation matrix (0.008622) confirmed suitability for factor analysis. Conflicting results emerged on the extraction of factors: Scree-plot indicated two or three factors (with two factors with an eigenvalue > 1 and one factor close to 1 (λ = 0.95)), Horn’s parallel analysis four factors, Velicer’s minimum average partial test two factors, and BIC three factors. All models were tested (see Table 3 for the results). The two-factor model accounted for 49% of the total variance, the three-factor model for 55%, and the four-factor model for 59%.

**Table 3:** Factor loadings and communalities for the EFA with two, three, and four factors – patient group

Item		Two-factor model						Three-factor model								Four-factor model								
		factor 1		factor 2		h^2^		factor 1		factor 2		factor 3		h^2^		factor 1		factor 2		factor 3		factor 4		h^2^
1. interruption of time		0.70		− 0.10		0.45		0.00		0.05		0.78		0.64		0.02		0.03		0.89		− 0.02		0.82
2. missing work		0.72		− 0.20		0.45		0.03		− 0.06		0.79		0.63		0.09		− 0.05		0.58		0.28		0.57
3. financial strain		0.60		− 0.11		0.33		0.25		− 0.04		0.40		0.33		0.17		− 0.01		0.23		0.51		0.49
4. disruption of relationships		0.62		0.19		0.51		0.49		0.19		0.18		0.51		0.51		0.19		0.12		0.10		0.51
5. sad		0.63		0.09		0.44		0.90		− 0.03		− 0.14		0.66		0.83		0.00		− 0.18		0.12		0.63
6. embarrassed		0.05		0.59		0.38		− 0.08		0.65		0.09		0.42		− 0.11		0.68		0.04		0.16		0.45
7. angry		0.02		0.88		0.78		0.03		0.88		− 0.05		0.77		0.06		0.87		0.00		− 0.10		0.79
8. worried		0.61		0.04		0.40		0.54		0.02		0.14		0.41		0.52		0.04		0.03		0.22		0.43
9. resentful		0.01		0.61		0.37		0.02		0.61		− 0.05		0.37		0.03		0.60		− 0.07		0.05		0.37
10. tired		0.76		0.06		0.61		0.70		0.03		0.16		0.65		0.81		− 0.01		0.15		− 0.14		0.74
11. toll on family		0.80		0.10		0.71		0.56		0.13		0.31		0.70		0.61		0.11		0.27		0.01		0.71
Explained variance (in %)		34		15				23		16		17				24		16		14		6		

### CFA – patient group

In the next step, all models were tested with a CFA. As the factorial structure of the three-factor model did not fully correspond to the original study, we also examined the fit of the original three-factor model.

**Fig. 1:**
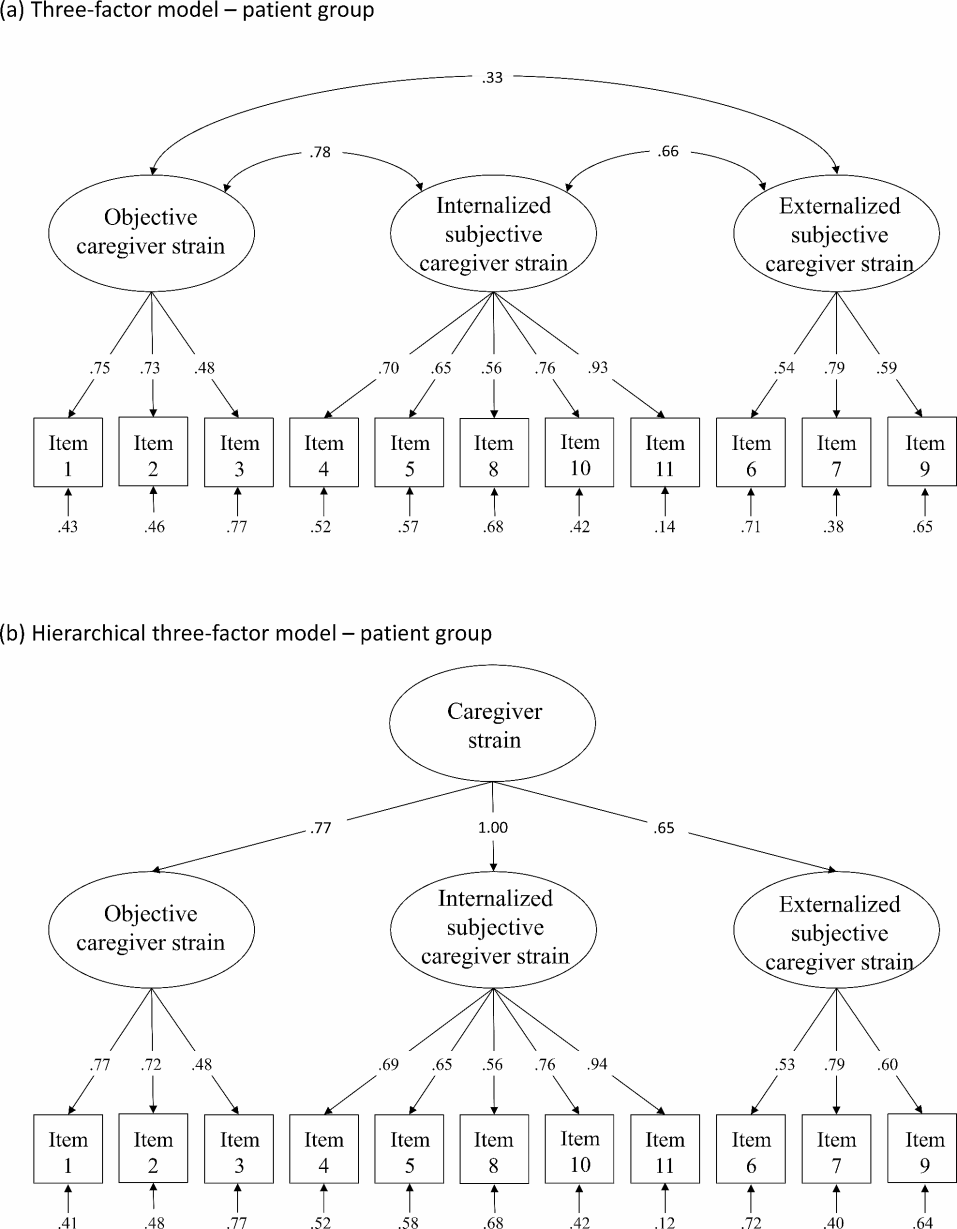
**Path diagram and estimates of the three-factor model and the hierarchical three-factor model in the patient group.** Ovals represent the latent constructs, rectangles represent the observed variables. Factor loadings and error variances are standardized

Results are presented in Table 4. Only the four-factor model (except for the *p*-value) and three-factor model found in the EFA showed good to acceptable values of fit indices. As the *χ*^2^-difference test was not significant (Δ*χ*^2^ = 1.37, Δ*df* = 2, *p* = .50), the less restrictive and more parsimonious three-factor model was preferred. The final model is presented in Fig. 1a.

**Table 4:** Goodness-of-fit indices – CFA

	SBχ2	df	*P*	CFI	RMSEA^a^	SRMR
Patient group						
Two-factor model	113.15	43	< 0.001	0.915	0.089 [0.070, 0.110]	0.065
Three-factor model – EFA	71.32	41	0.002	0.963	0.060 [0.036, 0.083]	0.050
Three-factor model – original study	106.88	41	< 0.001	0.922	0.088 [0.067, 0.108]	0.063
Four-factor model	69.95	39	0.002	0.962	0.062 [0.038, 0.086]	0.049
Healthy group						
Two-factor model	82.08	43	< 0.001	0.934	0.093 [0.059, 0.125]	0.061
Three-factor model	69.82	41	0.003	0.952	0.081 [0.045, 0.115]	0.058
Modified three-factor model	55.43	40	0.053	0.975	0.059 [0.000, 0.097]	0.039

*Notes.* SB = Satorra-Bentler scaled statistic. CFI = Comparative Fit Index. RMSEA = Root Mean Square Error of Approximation. SRMR = Standardized Root Mean Square Residual.

^a^RMSEA with 90%-confidence interval

### EFA – healthy group

Means, standard deviations, range, skewness, and kurtosis are shown in Table 2. Except for item #3 (kurtosis), values were within the critical thresholds [51]. Due to an acceptable overall picture of skewness and kurtosis and the use of robust estimators, no transformation was necessary.

The Bartlett’s test of sphericity (*χ*^2^(55) = 705.850, *p* < .001), the KMO measure (MSA = 0.87), and the determinant of the correlation matrix (0.004858) supported the adequacy of factor analysis. The Scree-plot, Velicer’s minimum average partial test, and the Bayesian Information Criterion suggested the extraction of two factors, whereas eigenvalues > 1 and Horn’s parallel analysis supported the extraction of three factors.

The EFA results are presented in Table 5. The two-factor model accounted for 50% of the total variance, the three-factor model for 54%. Because the pattern of factor loadings lacked interpretability, we did not consider the three-factor model for further analyses.

**Table 5:** Factor loadings and communalities for the EFA with two, three, and four factors – healthy group

Item		One-factor model				Three-factor model			
		factor 1	factor 2	h^2^		factor 1	factor 2	factor 3	h^2^
1. interruption of time		0.19	0.67	0.61		0.05	0.02	0.75	0.60
2. missing work		− 0.08	0.92	0.78		− 0.06	− 0.16	0.94	0.75
3. financial strain		0.13	0.25	0.11		0.28	− 0.18	0.32	0.17
4. disruption of relationships		0.66	0.15	0.56		0.31	0.31	0.33	0.55
5. sad		0.46	0.40	0.56		0.25	0.14	0.55	0.57
6. embarrassed		0.54	− 0.16	0.23		0.72	0.01	− 0.08	0.49
7. angry		0.80	− 0.07	0.58		0.11	0.69	0.09	0.64
8. worried		0.33	0.13	0.17		0.32	0.02	0.22	0.20
9. resentful		0.66	− 0.26	0.33		0.05	0.65	− 0.14	0.39
10. tired		0.46	0.52	0.73		− 0.09	0.41	0.68	0.80
11. toll on family		0.68	0.35	0.82		0.28	0.32	0.54	0.81
Explained variance (in %)		29	21			28	15	11	

### CFA – healthy group

Both the two-factor model and the three-factor model of the original study were each tested in a CFA (see Table 4). A significant *χ*^*2*^-difference test (Δ*χ*^2^ = 12.25, Δ*df* = 2, *p* = .002) suggested giving preference to the three-factor model.

**Fig. 2:**
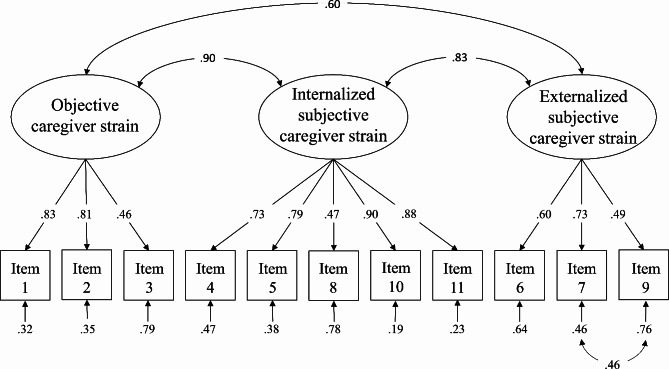
**Path diagram and estimates of the three-factor model in the healthy group.** Ovals represent the latent constructs, rectangles represent the observed variables. Factor loadings and error variances are standardized

To improve the model fit, we analyzed the modification indices. We successively allowed item #4 (“Is there disruption or upset of relationships within the family due to your child’s problems?”) to load on internalized subjective CS, and error terms of item #7 (“How angry do you feel towards your child?”) and item #9 (“How resentful do you feel towards your child?”) to correlate. As a result, the loading of item #4 on objective CS decreased (λ = -0.04), so this path was removed. The final model is presented in Fig. 2.

### Measurement invariance

The CFAs resulted in similar models for both groups, only differing in the error term correlation. Since this modification was also suggested in the patient group, models seem to be comparable. A test of invariance across parents of children with and without SHCN appeared to be appropriate.

The configural invariance model showed an acceptable fit. Concerning metric invariance, the changes in goodness-of-fit measures were above the critical thresholds (see Additional File 2: Table S1). Consequently, we cannot assume equal factor loadings in both groups.

### Second-order CFA – patient group

As we hypothesized a superordinate factor, named CS, that allows the use of a global score, we tested a hierarchical second-order model based on the three-factor model in the patient group. Except for an unsatisfactory *p*-value, all goodness-of-fit indices were good to acceptable (*χ*^2^ = 80.22, *df* = 41, *p* < .001, CFI = 0.952, RMSEA = 0.069 [0.046, 0.091], SRMR = 0.055). The model is presented in Fig. 1b.

### Second-order CFA –healthy group

We also examined a hierarchical model with CS as second-order factor and objective, internalized subjective, and externalized subjective strain in accordance with the original model as first-order factors in the healthy group. Despite a satisfactory global fit (*χ*^2^ = 60.16, *df* = 40, *p* = .02, CFI = 0.966, RMSEA = 0.070 [0.028, 0.104], SRMR = 0.043), a non-significant error variance of factor 1 suggested specification errors.

### Reliability

Internal consistencies and test-retest correlations reached largely satisfactory values in both groups (see Table 6). In addition, McDonald’s omega reached values not significantly lower than 0.70 both in the patient group (global strain: ω_h_ = 0.81; objective strain: ω_T_ = 0.70; internalized subjective strain: ω_T_ = 0.85; externalized subjective strain: ω_T_ = 0.68), and in the healthy group (objective strain: ω_T_ = 0.75; internalized subjective strain: ω_T_ = 0.87; externalized subjective strain: ω_T_ = 0.75).

**Table 6:** Internal consistencies and test-retest reliability of the German CGSQ-SF11

		Patient group								Healthy group						
		M (SD)		Range		Cronbach’s α^a^		ICC^a^		*M* (SD)		Range		Cronbach’s α^a^		ICC^a^
Objective strain		3.02 (0.92)		1–5		0.72 [0.67, 0.76]		0.86 [0.82, 0.90]		2.02 (0.85)		1-4.33		0.83 [0.80, 0.86]		0.81 [0.73, 0.87]
Internalized subjective strain		3.37 (0.91)		1–5		0.85 [0.83, 0.87]		0.91 [0.86, 0.94]		2.12 (0.93)		1–5		0.85 [0.82, 0.88]		0.88 [0.83, 0.92]
Externalized subjective strain		1.65 (0.74)		1-4.67		0.69 [0.63, 0.74]		0.88 [0.84, 0.91]		1.49 (0.6)		1–4		0.66 [0.58, 0.72]		0.82 [0.74, 0.87]
Global strain		2.80 (0.71)		1.09–4.64		0.86 [0.84, 0.88]		0.92 [0.89, 0.94]		n.a.		n.a.		n.a.		n.a.

Note. ICC = intraclass correlation coefficient. n.a. = not applicable.

^a^Cronbach’s α and ICC with 95%-confidence interval

### Convergent and divergent validity

The correlations of the three factors of the CGSQ-SF11, the AVE, the MSV, and the ASV are presented in Table 7. Furthermore, CS showed medium to high correlations with parenting stress, perceived stress, anxiety, depression, and family-related quality of life, confirming convergent validity in both groups. In contrast, it showed only low correlations with social desirability, indicating divergent validity (see Table 8).

**Table 7:** Factor correlations of the CGSQ-SF11, average variance extracted, maximum shared variance, and average shared variance

	Patient group							Healthy group					
	AVE	MSV	ASV		Factor correlation			AVE	MSV	ASV		Factor correlation	
					(1)	(2)						(1)	(2)
(1) Objective	0.44	0.59	0.42					0.52	0.83	0.54			
(2) Internalized subjective	0.53	0.59	0.50		.77^b^			0.59	0.83	0.65		.91^b^	
(3) Externalized subjective	0.42	0.41	0.33		0.50	0.64		0.51	0.46	0.36		0.50	0.68
(4) Global strain	.36^a^	-	-		-	-		-	-	-		-	-

Note. AVE = average variance extracted. MSV = maximum shared variance. ASV = average shared variance.

^a^ indicates an AVE significantly lower than 0.5.

^b^ indicates AVE is significantly less than the squared-correlation

**Table 8:** Correlations between CGSQ-SF11 and validation measures

		Patient group								Healthy group				
		CGSQ – global		CGSQ – objective		CGSQ – ext. subj		CGSQ – int. subj.		CGSQ – objective		CGSQ – ext. subj		CGSQ – int. subj.
CGSQ – objective		0.76**		—						—				
CGSQ – ext. subj.		0.64**		0.21**		—				0.50**		—		
CGSQ – int. subj.		0.93**		0.59**		0.47**		—		0.88**		0.61**		—
PSI-SF		0.75**		0.48**		0.64**		0.67**		0.58**		0.63**		0.65**
PSS-10		0.59**		0.39**		0.36**		0.60**		0.40**		0.31**		0.43**
GAD-7		0.55**		0.36**		0.33**		0.55**		0.38**		0.26**		0.45**
PHQ-8		0.51**		0.34**		0.29**		0.52**		0.36**		0.22**		0.42**
FLQ		− 0.52**		− 0.38**		− 0.27**		− 0.52**		− 0.45**		− 0.29**		− 0.45**
SES-17		− 0.06		− 0.03		− 0.16**		0.00		− 0.10*		− 0.09		− 0.02

Note. CGSQ = Caregiver Strain Questionnaire. Ext. subj. = external subjective strain. Int. subj. = internal subjective strain. PSI-SF = Parenting Stress Index. PSS-10 = Perceived Stress Scale-10. GAD-7 = Generalized Anxiety Disorder Screener. PHQ-8 = Patient Health Questionnaire. FLQ = Family-related Life Quality Questionnaire. SES-17 = Social Desirability Scale-17.

* indicates *p* < .05. ** indicates *p* < .01

### Incremental validity

Results of hierarchical regressions are presented in Additional File 2: Table S2. The model predicted 46% of the variance in stress, 40% of the variance in anxiety, 40% of the variance in depression, and 43% of the variance in family-related quality of life in the patient group. Examining only the variance accounted by the second step, the CGSQ-SF11 predicted an additional 7% of the variance in stress and anxiety and 5% in depression and family-related quality of life, respectively. Concerning the healthy group, the model predicted 43% of the variance in stress, 39% in anxiety, 46% in depression, and 56% in family-related quality of life. The CGSQ-SF11 predicted an additional 4% of the variance in anxiety, 5% in depression, and 2% in family-related quality of life.

### Floor and ceiling effects

Possible scores on all scales ranged between 1 and 5. No ceiling effects were present, with 0 − 3.80% of participants in the patient group reaching the highest score on the subscales or the global score, respectively, and 0 − 0.36% in the healthy group. In terms of floor effects, 0.48% of the caregivers in the patient group attained a score of 1 for the objective strain as well as the internalized subjective strain, 36.34% for the externalized subjective strain, and none for the global score. Concerning the healthy group, 20.94% reached the minimal score for objective strain, 15.52% for internalized subjective strain, 42.96% for externalized subjective strain, and 14.80% for subjective strain.

### Common method bias

The Harman’s single factor test showed that one factor accounted for 25% of the total variance, indicating no common method bias. Also, the addition of a latent method factor did not significantly improve the fit of a model including the CGSQ-SF11, PSI-SF, PSS-10, GAD-7, PHQ-8, FLQ, and SES-17 (Δχ2 = 0.135, Δdf = 2, *p* > .99).

## Discussion

The CGSQ-SF11 as a short form of the commonly used CGSQ is an efficient instrument to assess CS [5]. This study aimed to provide a German version of the scale, and to examine its psychometric properties including factorial validity, reliability, and construct validity.

Suggesting a two-, three-, or four-factor structure, our EFAs revealed ambiguous results in the patient group. In accordance with the original study [5], as well as other studies examining the long form of the CGSQ [1, 15, 16, 20], our CFA confirmed a three-factor model including objective, internalized subjective, and externalized subjective strain. Although the factor loading of item #4 (“Is there disruption or upset of relationships within the family due to your child’s problems?”) on the internalized subjective CS rather than objective CS is not consistent with previous versions, it is in line with Brannan et al. [1], who determined an equivalent cross-loading for this item. The overall pattern of factor loadings was meaningful.

In terms of scoring the various forms of the CGSQ, many studies use a global score [18]. This also applies to the CGSQ-SF11, which shows an acceptable fit for a one-factor model [5]. Our study added the examination of a second-order CFA, which is more appropriate to investigate the assumed hierarchical factor pattern, allowing the use of a global score [51, 52].

Furthermore, our study extended previous research by examining the applicability of the CGSQ-SF11 to parents of children without SHCN. To date, only Yang et al. [16] in China have investigated the use of the CGSQ among parents of typically developed children. They found little support for its suitability in this group and assumed a lack of seriosity in answering the questionnaire in this subsample. A short form of the questionnaire may overcome this problem. Furthermore, few missing values indicate high acceptance in our cohort. The EFAs in the healthy group revealed ambiguous results, suggesting a one- and a three-factor model. Because the pattern matrix of the three-factor model lacked content-related interpretability, this model was rejected. Instead, our CFA confirmed the three-factor structure of the original scale with a meaningful overall pattern of factor loadings. However, to receive an acceptable fit, error term correlation was allowed for item #7 and item #9. Both items refer to strong negative feelings towards the child. Difficulties in acknowledging and accepting the feelings [53] support the validity of the error-term correlation. Our results regarding the healthy group are in contrast to those of Yang et al. [16]. One explanation could be, that unlike Yang et al. [16], we applied modification indices. Of note, ASVs slightly higher than AVEs indicated that the objective strain and internalized subjective strain do not discriminate highly in this group.

Measurement invariance between groups was not supported indicating that the questionnaire measures distinct constructs in both groups, and comparisons are not possible. In order to capture additional burdens exceeding the common demands of parenting, the items of the CGSQ-SF11 refer to “the child’s problems”. Based on the unique challenges of caring for a child with SHCN [2], we assume the term to be associated with different aspects of life, and especially different demands in both groups. In concordance, the results of the two groups show different incremental validity. While CS predicts 5 − 7% of unique variance in stress, anxiety, depression, and family-related quality of life above and beyond the demographic variables and parenting stress in the patient group, this is not the case for stress in the healthy group. The proportion of unique variance in the remaining variables is lower than in the patient group. These results suggest that the construct of CS is slightly closer to parenting stress for parents of children without SHCN than in the patient group.

Floor effects appeared in all three subscales of the questionnaire in the healthy group, indicating that the questionnaire primarily discriminates between participants with high CS. Therefore, the CGSQ-SF11 is recommended to be administered mainly in strained populations or as a screening tool in the general population. The tendency to report more internalized subjective strain than externalized subjective strain is consistent with previous studies investigating the CGSQ and CGSQ-SF11 [5, 16, 20].

As expected, the German version of the CGSQ-SF11 yielded significant medium to high correlations with a range of related constructs supporting convergent validity. Our results replicated evidence that CS is associated with deteriorated mental health and a lower quality of life [3, 8]. The strongest associations were found with parenting stress. High correlations are in line with the literature [20] and demonstrate the conceptual overlap of both constructs. Interestingly, internal subjective strain showed the strongest correlations with all convergent constructs. With respect to this pattern, previous research revealed conflicting findings [e.g. 1, 23]. Our results reflect the internalized character of perceived stress, anxiety, and depression. Also, divergent validity was confirmed by low correlations with social desirability.

In terms of reliability, the objective and internalized subjective strain demonstrated good to adequate internal consistencies in both groups. Although at least adequate, lower values for the external subjective strain subscale are in line with the existing literature [5, 18]. ICCs demonstrated good to excellent test-retest reliability in both groups, supporting the temporal stability of the construct.

### Strengths, limitations, and future implications

The results of our study should be considered in light of its strengths and limitations. To begin with the strengths, one should first mention the thorough translation process according to WHO guidelines. Second, we pursued a two-step analytic strategy with an EFA followed by a CFA to explore the factor structure. This approach complies with best-practice guidelines [36]. Third, to overcome limitations of previous studies leaving aside the hierarchical structure of the questionnaire, we conducted a second-order CFA confirming the use of a global score. Fourth, we were able to conduct an extensive validation comprising important related and distinct constructs. Assessing CS at two time points, enabled us to determine test-retest reliability. Fifth, we included a diverse sample, allowing us to examine the applicability and measurement invariance of the questionnaire in a healthy subsample.

With respect to the limitations of our study, two aspects should be noted. First, we collected a convenience sample, which may underlie a self-selection bias. Our sample mainly consists of mothers and cannot be expected to be representative of the German population. As for the sample of fathers, our results have to be generalized with caution. However, we assume differences between mothers and fathers in the level of CS [5], but not regarding the factor structure of our questionnaire. Second, we modified the model in the healthy group using modification indices to attain a good fit. Although we based our decision on theory, this adds an exploratory component to our confirmatory analyses. Therefore, future research should cross-validate the CGSQ-SF11 in this subsample [54] to validate our results. Finally, future research is warranted to replicate our results in adequate-sized, representative samples that allow to test for measurement invariance across socio-demographic characteristics and to provide cut-off scores and norm values for the German CGSQ-SF11.

## Conclusions

To conclude, the German CGSQ-SF11 has been demonstrated to be a reliable and valid measure of CS capturing the objective, internalized subjective, and externalized subjective strain. Given the impact of CS on both parental and child mental health, the use of this instrument in practice could be recommended for the early identification of families with high burden to provide prevention and intervention services. Its use in future research will enable researchers to examine the different dimensions of CS with an efficient instrument in German-speaking countries among parents of children with and without SHCN.

### Electronic supplementary material

Below is the link to the electronic supplementary material.

Supplementary Material 1

Supplementary Material 2

## Data Availability

The datasets generated and analyzed during the current study are not publicly available, but are available from the corresponding author on reasonable request.

## Abbreviations

BIC: Bayesian Information Criterion
CFA: Confirmatory factor analysis
CFI: Comparative Fit Index
CGSQ: Caregiver Strain Questionnaire
CGSQ-SF11: Caregiver Strain Questionnaire-Short Form 11
CS: Caregiver strain
EFA: exploratory factor analysis
FIML: Full information maximum likelihood
FLQ: Family-related Life Quality Questionnaire
GAD-7: Generalized Anxiety Disorder Screener
ICC: Intraclass correlation coefficient
KMO: Kaiser-Meyer-Olkin measure of sampling adequacy
PAF: Principal axis factoring
PHQ-8: Patient Health Questionnaire
PSI-SF: Parenting Stress Index
PSS-10: Perceived Stress Scale-10
RMSEA: Root Mean Square Error of Approximation
SB: Satorra-Bentler scaled statistic
SES: Socioeconomic status
SES-17: Social desirability scale-17
SHCN: Special healthcare needs
SRMR: Standardized Root Mean Square Residual
