# Supplementary material for: Validation of a German version of the caregiver strain questionnaire-short form 11 (CGSQ-SF11)
Source: BMC Psychol. 2024 Jul 10;12:386. doi: 10.1186/s40359-024-01875-7 (PMC11234714; doi:10.1186/s40359-024-01875-7)
Supplement: Supplementary file 1 — Supplementary Material 1 [file 40359_2024_1875_MOESM1_ESM.pdf]

Additional File 1

Original items of the Caregiver Strain Questionnaire – Short Form 11 (CGSQ-SF11) and their German translation

Original items		German translation	
1.	Do your child's problems interrupt your personal time?	1.	Unterbrechen die Probleme Ihres Kindes die Zeit, die Sie für sich selbst haben?
2.	Do you miss work or neglect other duties because of your child's problems?	2.	Fehlen Sie bei der Arbeit oder vernachlässigen Sie andere Pflichten aufgrund der Probleme Ihres Kindes?
3.	Do your child's problems place a financial strain on your family?	3.	Stellen die Probleme Ihres Kindes eine finanzielle Belastung für Ihre Familie dar?
4.	Is there disruption or upset of relationships within the family due to your child's problems?	4.	Sind die Beziehungen innerhalb der Familie aufgrund der Probleme Ihres Kindes gestört oder erschüttert?
5.	How sad or unhappy do you feel as a result of your child's problems?	5.	Wie traurig oder unglücklich fühlen Sie sich als Folge der Probleme Ihres Kindes?
6.	How embarrassed do you feel about your child's problems?	6.	Wie peinlich sind Ihnen die Probleme Ihres Kindes?
7.	How angry do you feel toward your child?	7.	Wie verärgert fühlen Sie sich über Ihr Kind?
8.	How worried do you feel about your child's future?	8.	Wie besorgt fühlen Sie sich um die Zukunft Ihres Kindes?
9.	How resentful do you feel toward your child?	9.	Wie missgünstig sind Sie Ihrem Kind gegenüber?
10.	How tired or strained do you feel as a result of your child's problems?	10.	Wie müde oder angespannt fühlen Sie sich als Folge der Probleme Ihres Kindes?
11.	In general, how much of a toll do your child's problems take on your family?	11.	Im Allgemeinen, wie sehr sind die Probleme Ihres Kindes eine Last für Ihre Familie?
