# Supplementary material for: Validation of a German version of the caregiver strain questionnaire-short form 11 (CGSQ-SF11)
Source: BMC Psychol. 2024 Jul 10;12:386. doi: 10.1186/s40359-024-01875-7 (PMC11234714; doi:10.1186/s40359-024-01875-7)
Supplement: Supplementary file 2 — Supplementary Material 2 [file 40359_2024_1875_MOESM2_ESM.pdf]

**Additional File 2**

**Table S1.** Measurement invariance across patient group and healthy group

| Model | SB$\chi^2$ | *df* | CFI | RMSEA | SRMR | Model Comparison | $\Delta\chi^2$ | $\Delta$*df* | $\Delta$CFI | $\Delta$RMSEA[a] | $\Delta$SRMR | *p* |
|---|---|---|---|---|---|---|---|---|---|---|---|---|
| Configural | 115.624 | 80 | .974 | .055 [.030, .076] | .041 | | | | | | | |
| Metric | 159.509 | 88 | .948 | .074 [.055, .092] | .074 | Configural vs. Metric | 43.885 | 8 | .026 | .019 | .033 | <.001 |

*Note*. SB = Satorra-Bentler scaled statistic. CFI = robust Comparative Fit Index. RMSEA = robust Root Mean Square Error of Approximation. SRMR = robust Standardized Root Mean Square Residual.

[a]RMSEA with 90%-confidence interval

**Table S2.** Incremental variance in stress, anxiety, depression, and family-related quality of life (standardized beta values)

| | Patient group | | | | Healthy group | | | | |
|---|---|---|---|---|---|---|---|---|---|
| Outcome variable | PSS | GAD | PHQ | FLQ | | PSS | GAD | PHQ | FLQ |
| Step 1 | $R^2$ = .387 | $R^2$ = .333 | $R^2$ = .343 | $R^2$ = .378 | Step 1 | $R^2$ = .416 | $R^2$ = .354 | $R^2$ = .418 | $R^2$ = .536 |
| Caregiver’s age | -.034 | -.010 | -.048 | -.060 | Caregiver’s age | -.008 | -.010 | -.049 | -.121 |
| Caregiver‘s sex (m)[a] | .075 | -.020 | .010 | .149 | Caregiver‘s sex (m)[a] | -.030 | .112 | .083 | .330 |
| Caregiver’s sex (f)[a] | .129 | .049 | .101 | .069 | Caregiver’s sex (f)[a] | -.006 | .118 | .111 | .288 |
| Child’s age | -.042 | -.070 | -.001 | .166** | Child’s age | -.023 | -.006 | .003 | .290*** |
| Child’s sex (m)[a] | .445 | .701 | .228 | -.004 | | | | | |
| Child’s sex (f)[a] | .393 | .688 | .326 | .073 | Child’s sex | -.047 | -.103 | -.130* | .117* |
| SES | -.107* | -.137** | -.148*** | .218*** | SES | -.148** | -.154** | -.241*** | .179*** |
| PSI-SF | .583*** | .511*** | .520*** | -.488*** | PSI-SF | .609*** | .547*** | .543*** | -.610*** |
| Step 2 | $R^2$ = .461 $\Delta R^2$ = .074*** | $R^2$ = .402 $\Delta R^2$ = .069*** | $R^2$ = .397 $\Delta R^2$ = .054*** | $R^2$ = .431 $\Delta R^2$ = .053*** | Step 2 | $R^2$ = .427 $\Delta R^2$ = .011 | $R^2$ = .391 $\Delta R^2$ = .037*** | $R^2$ = .466 $\Delta R^2$ = .048*** | $R^2$ = .555 $\Delta R^2$ = .019* |
| Caregiver’s age | -.046 | -.018 | -.050 | -.055 | Caregiver’s age | -.031 | -.048 | -.094 | -.095 |
| Caregiver‘s sex (m)[a] | .096 | .001 | .024 | .136 | Caregiver‘s sex (m)[a] | -.043 | .069 | .056 | .320 |
| Caregiver’s sex (f)[a] | .119 | .040 | .098 | .074 | Caregiver’s sex (f)[a] | -.009 | .098 | .104 | .267 |
| Child’s age | -.056 | -.082 | -.015 | .181*** | Child’s age | .013 | .017 | .048 | .250*** |
| Child’s sex (m)[a] | .539 | .789* | .424 | -.103 | | | | | |
| Child’s sex (f)[a] | .520 | .809* | .438 | -.053 | Child’s sex | -.037 | -.098 | -.118* | .108* |
| SES | -.091* | -.118** | -.142*** | .209*** | SES | -.145** | -.134* | -.229*** | .176*** |
| PSI-SF | .393*** | .340*** | .465*** | -.431*** | PSI-SF | .635*** | .525*** | .586*** | -.623*** |
| CGSQ – objective | -.031 | -.017 | -.047 | .031 | CGSQ – objective | .029 | -.058 | -.034 | -.058 |
| CGSQ – ext. Subj. | -.084 | -.082 | -.166** | .180*** | CGSQ – ext. Subj. | -.162** | -.234** | -.289*** | .175** |
| CGSQ – int. Subj. | .376*** | .344*** | .274*** | -.287*** | CGSQ – int. Subj. | .083 | .294* | .234 | -.090 |

*Note*. M = male. F = female. SES = socioeconomic status. PSI-SF = Parenting Stress Index. CGSQ = Caregiver Strain Questionnaire. Ext. subj. = external subjective strain. Int. subj. = internal subjective strain. PSS-10 = Perceived Stress Scale-10. GAD-7 = Generalized Anxiety Disorder Screener. PHQ-8 = Patient Health Questionnaire. FLQ = Family-related Life Quality Questionnaire.

[a]Sex was dummy-coded

* indicates $p$ < .05. ** indicates $p$ < .01. *** indicates $p$ < .001.
